# Mapping of the brain hemodynamic responses to sensorimotor stimulation in a rodent model: A BOLD fMRI study

**DOI:** 10.1371/journal.pone.0176512

**Published:** 2017-04-25

**Authors:** Salem Boussida, Amidou S. Traoré, Franck Durif

**Affiliations:** 1 UR370 QuaPA, INRA, Saint-Genès-Champanelle, France; 2 Neurologie-A, CHU, Clermont-Ferrand, France; University Paris 6, FRANCE

## Abstract

Blood Oxygenation Level Dependent functional MRI (BOLD fMRI) during electrical paw stimulation has been widely used in studies aimed at the understanding of the somatosensory network in rats. However, despite the well-established anatomical connections between cortical and subcortical structures of the sensorimotor system, most of these functional studies have been concentrated on the cortical effects of sensory electrical stimulation. BOLD fMRI study of the integration of a sensorimotor input across the sensorimotor network requires an appropriate methodology to elicit functional activation in cortical and subcortical areas owing to the regional differences in both neuronal and vascular architectures between these brain regions. Here, using a combination of low level anesthesia, long pulse duration of the electrical stimulation along with improved spatial and temporal signal to noise ratios, we provide a functional description of the main cortical and subcortical structures of the sensorimotor rat brain. With this calibrated fMRI protocol, unilateral non-noxious sensorimotor electrical hindpaw stimulation resulted in robust positive activations in the contralateral sensorimotor cortex and bilaterally in the sensorimotor thalamus nuclei, whereas negative activations were observed bilaterally in the dorsolateral caudate-putamen. These results demonstrate that, once the experimental setup allowing necessary spatial and temporal signal to noise ratios is reached, hemodynamic changes related to neuronal activity, as preserved by the combination of a soft anesthesia with a soft muscle relaxation, can be measured within the sensorimotor network. Moreover, the observed responses suggest that increasing pulse duration of the electrical stimulus adds a proprioceptive component to the sensory input that activates sensorimotor network in the brain, and that these activation patterns are similar to those induced by digits paw’s movements. These findings may find application in fMRI studies of sensorimotor disorders within cortico-basal network in rodents.

## Introduction

Several studies have been performed to investigate the organization and the neuronal connections of the sensorimotor system in the rat brain using histological tract-tracing [1,2] and electrophysiological mapping methods [3–5]. From these previous studies, a simplified description of the rat sensorimotor system, based on connections between the basal ganglia (BG), the thalamus and the sensorimotor cortex can be provided [1–7].

Functional Magnetic Resonance Imaging (fMRI) is widely used to study the brain function in response to peripheral stimulation, e.g. the somatosensory system [8–25]. However, a majority of these studies have concentrated on the cortical effects of sensory peripheral stimulation of the rat paw [8–10,12–23]. Moreover, sensory afferent projections are not limited to cortical areas of the rat brain but they are extended to neurons within the sensorimotor cortex, the thalamus, and the BG [26]. The lack of activations in subcortical areas such as BG and thalamus may come from different methodological issues, in stimulation-induced fMRI studies [27–31].

Firstly, fMRI in rodents is most often performed under general anesthesia. However, anesthesia directly affects cerebral blood flow [29] and metabolism [30] leading to alteration of the hemodynamic response to neural activity [32]. One of the most used anesthetics in fMRI studies in animals is isoflurane. This volatile agent shows potential for stimulation-induced studies under low level anesthesia [12].

Secondly, the vast majority of fMRI studies of paw representations in the rat brain [10,12–17,19–22] have been performed using a short pulse width (0.3–1ms). At these stimulus pulse widths, only the primary somatosensory cortex response has been reported [10,12–17,19–22] suggesting a merely somatosensory characteristic of the stimulus. An interesting study [23] shows that an increased stimulus pulse width alters the nature of a stimulus, resulting not only in sensory but also in proprioceptive input. This stimulus characteristic could be of interest while targeting subcortical structures, like the thalamus and the BG.

Thirdly, the small MR signal variation induced by brain activations in any fMRI study requires a necessary and sufficient Signal to Noise Ratio (SNR) to detect successfully these subtle changes [33]. This is critical for small and deeply located (subcortical) brain structures, where the required SNR is not always obtained with the widely used hardware configuration, including a surface coil for signal reception [27]. In addition, the ability of each fMRI protocol to reveal a relatively low signal change arising from BOLD effect is mainly determined by the resulting temporal signal to noise ratio (tSNR) of the time series [31]. This is particularly important when measuring BOLD signal changes across regions displaying heterogeneous neurovascular architecture [27] and consequently different noise characteristics. To ensure BOLD sensitivity to stimulation-induced signal changes in both cortical and subcortical structures, a sensitivity map depicting the minimum detectable signal change can be used [31].

In view of these potential methodological issues, the purpose of this study was to investigate BOLD fMRI of the rat sensorimotor network, using a calibrated imaging protocol, in response to a sensorimotor electrical stimulation of the rat paw, in non-painful conditions. Peripheral sensorimotor stimulation in rat fMRI should provide a useful tool to elicit functional activations in cortical and subcortical brain structures implicated in the integration of the sensorimotor information; thereby to investigate various pathologies with motor disorders.

## Materials and methods

### Animals

Twelve male adult *Wistar* rats (Charles River Laboratories, Paris-France) weighing 300 ± 20g were used in this study. They were housed at 20–22°C under a 12-hour light/12-hour dark cycle. All experiments were performed according to procedures conforming to European legislative, administrative and statutory regulations governing the protection of animals used for experimentation or other scientific purposes (86/609/EEC). The protocol was approved by the Regional Experimental Care and Use Committee (Auvergne CREEA). Every effort was made to minimize the number of animals used.

### Animal preparation for fMRI

Rats were initially anesthetized (induction) with 3% isoflurane and placed on a heated surgical table. During surgical preparation, isoflurane was reduced to 1.5–2% in a 70%/30% air/oxygen mixture, for maintenance during surgery. The caudal vein was cannulated for drug perfusion and the left femoral artery was cannulated with PE-50 tubing (Smiths Medical France) for arterial gas blood sampling access. Animals were intubated for mechanical ventilation (80 breaths/min, respiration cycle: 30% inhalation, 70% exhalation, CWE MRI ventilator) and then placed in a rodent stereotaxic device (Bruker, GmbH, Ettlingen, Germany) in a supine position. Before placing the rats in the magnet, 150 μl of Nimbex® (2mg/ml) was administered firstly in a bolus injection, through the caudal vein and then continuously (5mg/ml) administrated for a soft muscle relaxation (2.38mg/hr), coupled with a progressive isoflurane reduction to reach 0.7–0.8% concentration[34]. During imaging, body temperature, cardiac frequency and pulse oximetry parameters were continuously monitored (SA Instruments, Stony Brook, New York, USA). Arterial blood sampling (Osmetech OPTI CCA Blood gas analyzer): Oxygen saturation (SPO_2_), arterial pressure of oxygen (pO_2_), arterial pressure of carbon dioxide (pCO_2_) and potential hydrogen (pH) were achieved before and after each fMRI experiment.

### Electrical stimulation

Two copper needle electrodes were used for electrical stimulation. Electrodes were inserted subcutaneously in the palmar surface of the right hindpaw of each rat: one in the central axis of the proximal part of the hindpaw and the other in the lateral part of the paw. Electrical stimulation was obtained by delivering electrical square pulses using a Pulsar 6Bp bipolar stimulator (FHC Inc., Bowdoinham, ME, USA). Stimulation parameters were adapted from N. Van Camp et al. [23] and included current pulses with a 1.7 mA amplitude, 10 ms duration and 8 Hz frequency applied in a block-design. In a separate experiment outside the magnet, 1.7 mA-current intensity was found to be below the threshold of nociception (S1 Table), where no significant changes in cardiac frequency and pulse oximetry were observed while applying stimulation under our conditions of anesthesia [12,35,36]. As reported by N. Van Camp et al. [23] and observed in the present study, this set of electrical stimulation parameters resulted both in somatosensory and proprioceptive inputs owing to visible movement in the hindpaw's digits (S1 Video). A standard OFF/ON block design stimulation paradigm was then used during fMRI session starting with a resting period of 25s as a baseline followed by 25s of stimulation. Moreover, in order to compensate for small current intensity deviations during stimulation, real time current monitoring was carried out for each animal using a 54600B oscilloscope (HP Agilent, USA).

### Functional MR imaging

All imaging experiments were performed on a 4.7T Bruker (Biospec 47/40,Bruker, GmbH, Ettlingen, Germany) with a horizontal bore magnet equipped with a 12 cm gradient coil (Bruker BGA12, 400 mT/m) and interfaced to AVANCE III console. Two actively decoupled RF coils were used: a 7.2-cm diameter volume coil for transmission and a 2-cm diameter surface coil (Rapid Biomedical, Rimpar, Germany) positioned on the top of the animal's head for reception. After a rapid acquisition of pilot images using the standard Bruker tripilot protocol, a Turbo-RARE (rapid acquisition with relaxation enhancement) pulse sequence parameterized with an effective echo time (TE) of 33ms, a repetition time (TR) of 2777ms, a RARE factor of 16, a field of view (FOV) of 2.56 cm, and a matrix size of 256x256, to locate the volume of interest containing the sensorimotor cortex, the sensorimotor thalamic nuclei and the caudate-putamen areas at the magnet center was used. Magnetic field homogeneity was then optimized using the Bruker MAPSHIM shimming protocol. The procedure includes a whole brain shimming and a local shimming at first and second order shims to reach a 1H spectral full width at half maximum of 10–15 Hz. High resolution anatomical images were then acquired for brain normalization using a Turbo-RARE pulse sequence with the same acquisition parameter as above, except the RARE Factor of 8 and 2 averages. Ten 1-mm thick contiguous axial slices, from -6.36 mm to +2.64 mm to Bregma were acquired. Functional imaging was conducted at the same position of the high resolution images with a two-shot gradient echo planar imaging (GE-EPI) pulse sequence (2.56 cm^2^ FOV; 64x64 matrix size; a TR of 1000 ms; a flip angle of 50°) resulting in the pixel size of 0.4 mm^2^.

### Echo time

**Fig 1:**
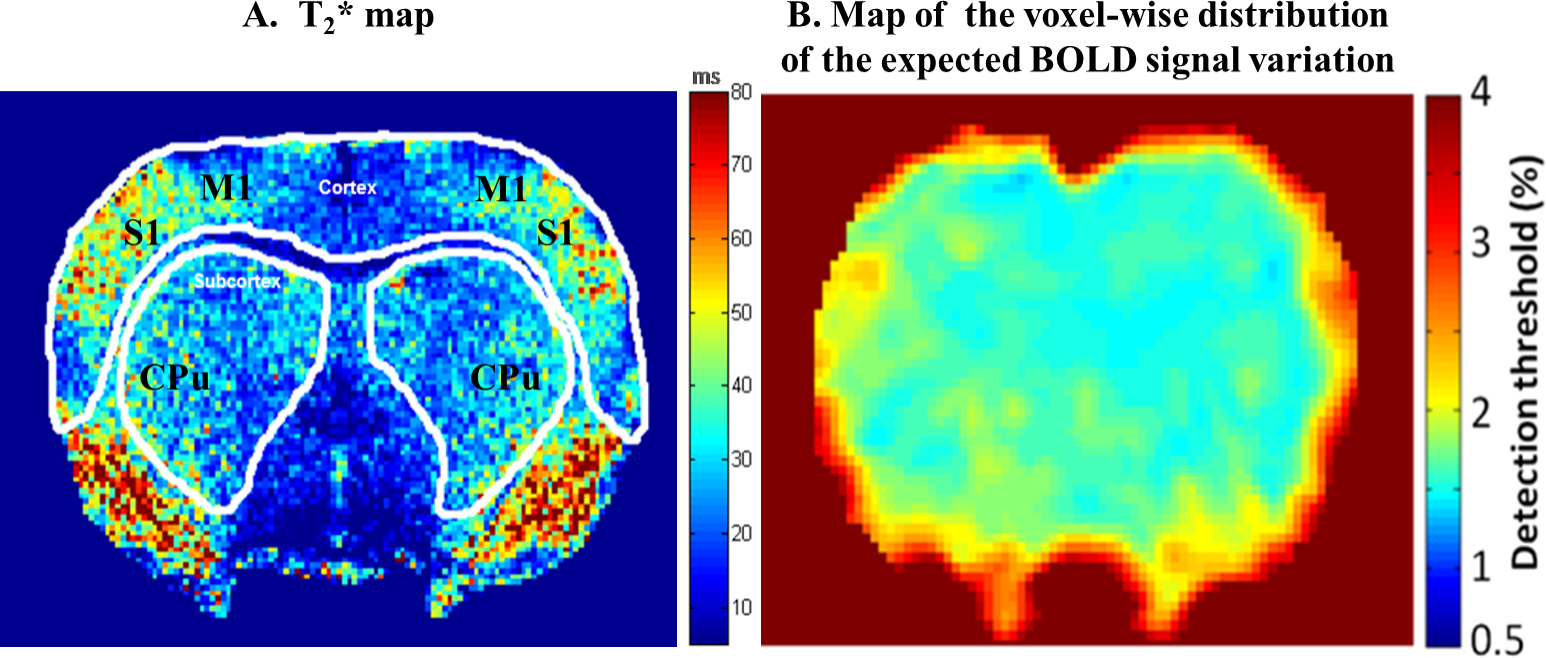
(A) Color coded map of the rat brain pixel-wise T2*. The mean T2* value of the region of interest (ROIs) drawn over cortex was ~40 ms (while mean T2* of primary sensory cortex (S1) was 37±5 ms) whereas for the subcortex region a mean T2* value of 30 ms was obtained. The caudate-putamen (CPu) and the primary motor cortex (M1), two sensorimotor structures, are presented for anatomical localization. **(B) Map of the voxel-wise distribution of the expected BOLD signal variation (%).** The voxel-wise map of the minimum BOLD signal variation (%) that can be measured under our experimental conditions is presented. The detection threshold is calculated for a significance level of p< 0.01.

**Fig 2:**
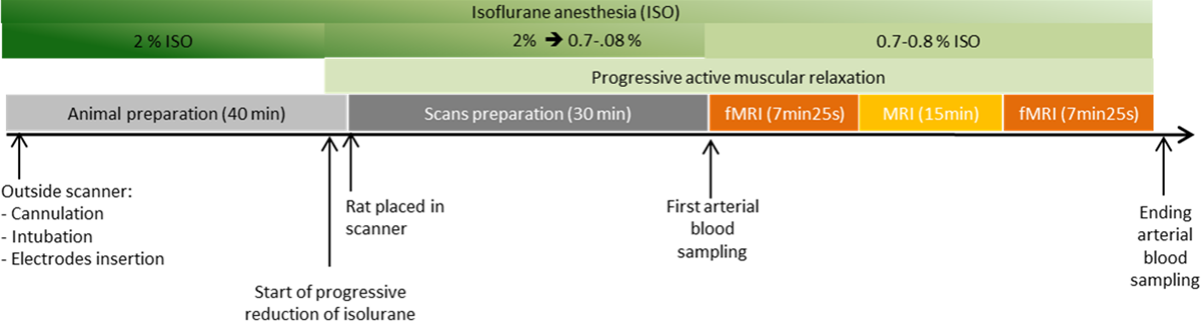
The experimental protocol. All the experiment steps are resumed, from animal preparation to functional imaging.

The echo time (TE) of the fMRI session was optimized to increase both BOLD sensitivity and specificity in the cortex and the subcortex. For this purpose in a separate experiment, the T2* map (Fig 1A) of the rat brain was constructed from the Gradient Echo Multi Echo (TR = 2000ms, 6 TE from 10 ms to 60ms- step 10 ms) image acquisition using the same position above and after shimming procedure. From these T2* maps, the mean T2* value of the region of interest (ROIs) drawn over cortex was ~40 ms (while mean T2* of primary sensory cortex (S1) was37±5 ms) whereas for the subcortex region a mean T2* value of 30ms was obtained. The TE of the pulse sequence plays a role in determining both sensitivity and specificity. Differentiation of the signal equation for a T2*-weighted acquisition, shows that the maximum effect will be observed when the TE is equal to the T2* value of the tissue compartment in question [37]. As the difference between the T2* values of these two regions were higher than 25%, we decided to perform two fMRI sessions, separated by 15 min for each animal (Fig 2). In the first session, the TE was set to 30 ms whereas 40 ms was used for the second.

### Block design and temporal SNR

To increase the sensitivity of our BOLD fMRI protocol, the number of blocks in the stimulation paradigm for each fMRI session was optimized to reach a minimum required temporal SNR (tSNR) in both cortical and subcortical regions of interest. For this purpose, theoretical model, tSNR = 2t/ΔS(N)^1/2^ [31], which describes the dependence of tSNR on the experimental parameters was used. Prior to the fMRI experiment, using the spatial SNR (sSNR) resulting from the above described fMRI protocol, the minimum number of scans (N) allowing the detection of an expected at least 1% BOLD signal change (ΔS) at the corresponding Student t test (p<0.01) was determined. For a sSNR of 40±3 in the subcortex (while 48±2 in the cortex) resulting from our experimental conditions, a minimum of 160 image acquisitions in the time series is required. To increase the statistical power within the fMRI time series [38], we decided to set the number of the repeated image acquisitions to 225. This condition was found to be a good compromise between a homogenous distribution of the minimum tSNR within cortical and subcortical brain regions (Fig 1B) as well as a stable physiological state of the animal. As a consequence, each fMRI session consisted of 225 GE-EPI image acquisitions, starting with 12 dummy scans to achieve the steady state magnetization, followed by 8 alternating rest/stimulation cycles (25s-OFF and 25s-ON) for a total acquisition time of 7min25s. Moreover, fMRI sessions were acquired with a 15 min rest period for neurovascular recovery before the next stimulation paradigm. The total duration of the overall experiment including animal preparation, scans preparation and fMRI scanning was 1 h 40 min on average (Fig 2).

### Functional MRI analysis

For each fMRI session, the first twelve EPI images were discarded from the analysis, to allow the MR signal to reach steady state. All processing steps were performed using the Statistical Parameter Mapping software (SPM8, the Welcome department of Cognitive Neurology, London) and MATLAB in-house programs. Pre-processed functional data (motion correction, segmentation, normalization to the rat stereotaxic Paxinos space and smoothing (three times the voxel size (0.4mm x 0.4mm x 1mm)) were then used to calculate single subject statistical t-maps using the General Linear Model (GLM, SPM8), where a pixel-by-pixel correlation between the fMRI signals and the stimulation paradigm is assessed. Motion parameters correction and the recorded cardiac frequency values were included within the statistical model as effects of no interest. Each fMRI session, i.e. TE of 30 ms and 40 ms was analyzed separately. For group mixed-effects analysis (n = 12), the derived statistical maps from the single subject GLM analysis of each session were used in a one-sample t-test. BOLD responses showing significant positive and negative correlations to the stimulus paradigm, were detected using a double statistical threshold [39] values of p = 0.01 (specificity) and p = 0.05 (sensitivity) for single and group analyses.

## Results

### Animal physiology

Under artificial respiration condition, all respiratory parameters were optimized to maintain an optimal physiological state of animals (Table 1). Pulse oximetry parameter (SPO_2_) was maintained at the level of 97–98% through the experiment by soft adjustments in the volume of the O_2_/N_2_ gas mixture inhalation. No significant differences in the physiological conditions were observed between the rest and the stimulation conditions for all animals (n = 12) (Table 1). Arterial blood gazes remained stable when comparing the start and the end of the experiment (Table 1) for all animals (n = 12).

**Table 1:** Physiological parameters.

Mean values	Complete fMRI experiment		Stimulation experiment	
	Start	End	Rest	Stimulation
FC (bpm)	399±12	410±16	403±14	414±13
SPO_2_ (%)	97±1	98±1	97±1	97±1
PaO_2_ (mmHg)	141±8	144±9		
PaCO_2_ (mmHg)	35±4	34±2		
Temperature (°C)	37±0.5	37±0.4	37±0.5	37±0.4

Data were recorded at the start and at the end of the fMRI experiment as well as under the two conditions paradigm (Rest/Stimulation) including mean cardiac frequency (FC), Oxygen saturation (SPO_2_), arterial pressure of oxygen (PaO_2_), arterial pressure of carbon dioxide (PaCO_2_) and mean temperature (°C) for 12 animals.

### BOLD maps

**Fig 3:**
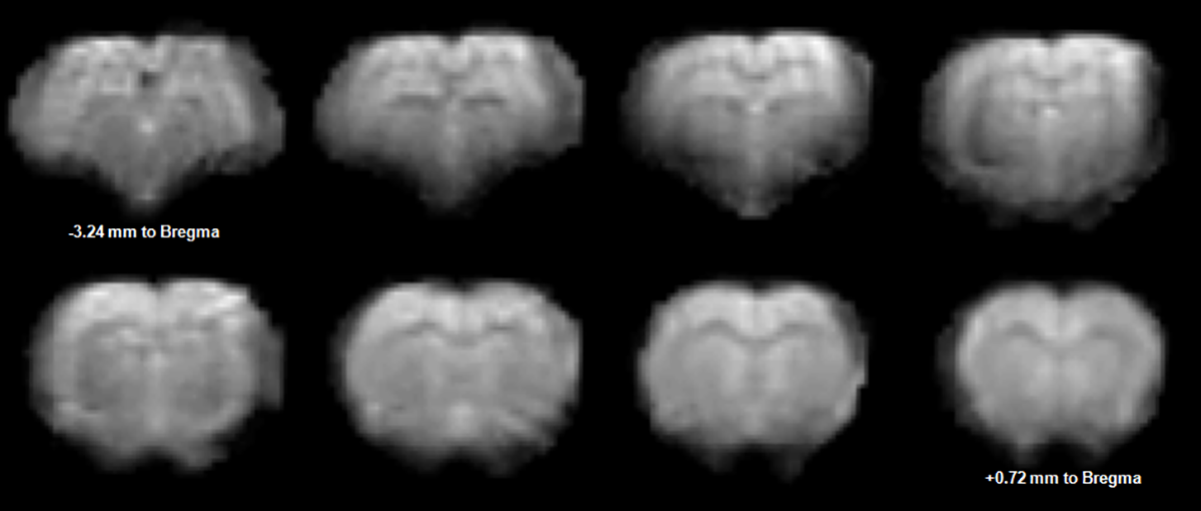
An example of a preprocessed (realignment, segmentation and spatial normalization) fMRI EPI image data set acquired with a TE of 30ms.

**Fig 4:**
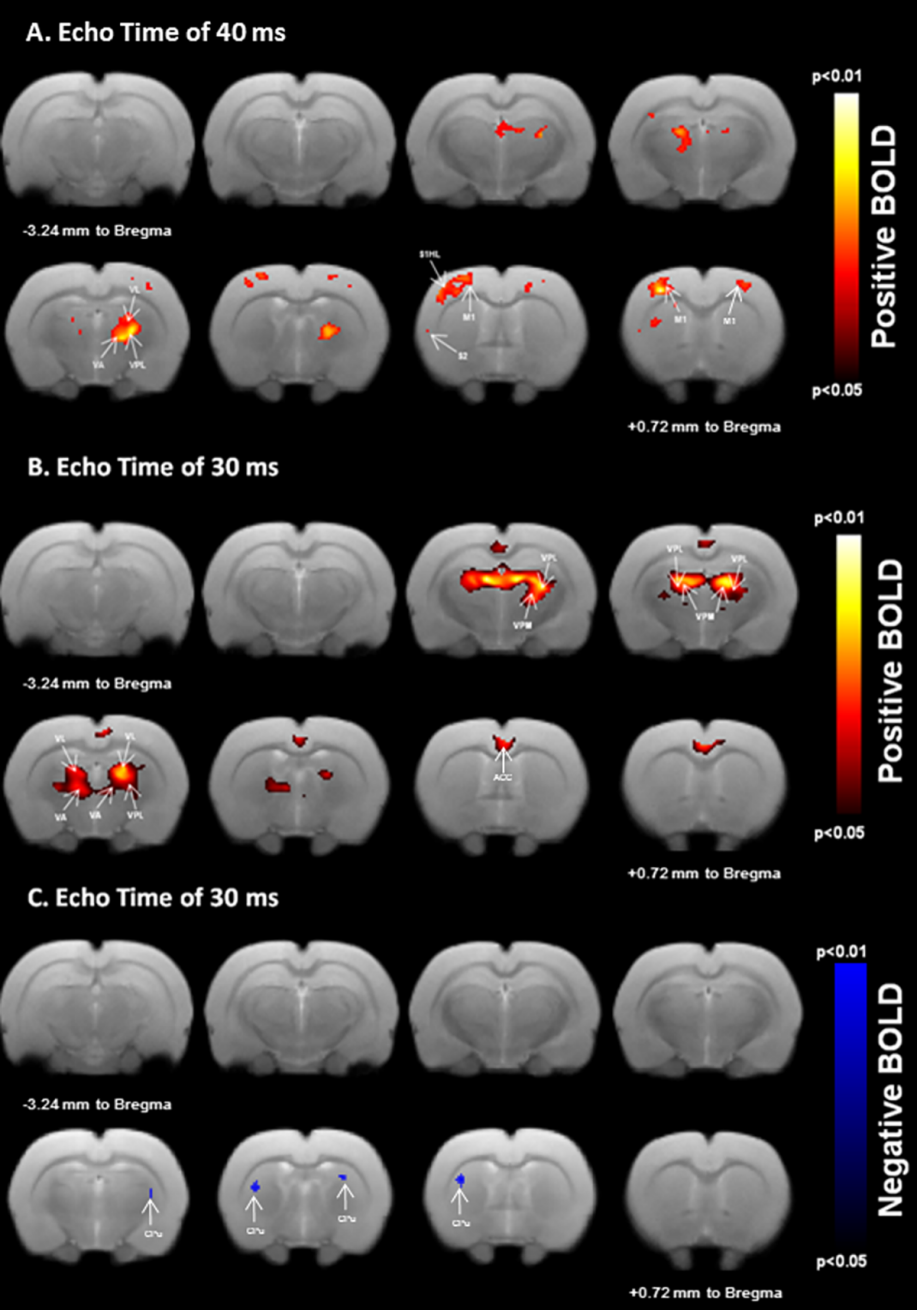
BOLD responses to unilateral sensorimotor electrical hindpaw stimulation. Statistical maps calculated from group analysis (n = 12; 0.05≤p-value≤0.01) are overlaid onto a template rat brain (eight axial slices centered at -3.24 mm to Bregma to +0.72 mm to Bregma). Unilateral stimulation resulted in positive (A and B) and negative (C) BOLD responses in cortical and subcortical regions relevant for sensorimotor processing. Positive activated areas include the contralateral primary/secondary hindpaw somatosensory (S1HL/S2), bilateral primary motor (M1) cortex (A) as well as the bilateral sensorimotor thalamic nuclei (VPL and VA/VL) at TE = 40ms (A), larger positive activations are localized in the bilateral sensorimotor thalamic nuclei (VPL and VA/VL) at TE = 30ms (B). Negative activations are bilaterally measured in the dorsolateral caudate-putamen (CPu) region at TE = 30ms (C).

Our fMRI protocol resulted in a good quality EPI images (Fig 3) allowing BOLD analysis. All datasets were included in the analysis since physiological parameters from all animals were within normal boundaries (Table 1) along with the tSNR whose values remained well above the minimal calculated value. The calculated statistical parametric maps were overlaid on the corresponding Turbo-RARE T2 images to identify better anatomical localization of BOLD signal changes. Eight slices centered from -3.24 mm (top left) to +0.72 mm to Bregma (bottom right) spanning the regions of interest are displayed in Fig 4. Positive BOLD responses occurred bilaterally in the sensorimotor thalamic nuclei. These activations were larger at TE = 30 ms than at TE = 40ms, as demonstrated by an increasing number of activated pixels in this structure (Fig 4A and Fig 4B, Table 2). The anatomic localizations of these activations were carefully identified by consulting the Paxinos rat brain atlas [6]. These responses were localized in the ventral posterior medial thalamic nucleus (VPM), the ventral posterior lateral thalamic nucleus (VPL), the ventral anterior thalamic nucleus (VA) and the ventral lateral thalamic nucleus (VL). As can be seen in Fig 4A, electrical stimulation elicited a contralateral BOLD response localized in the sensorimotor cortex only at TE = 40ms. Both the primary and the secondary sensory hindpaw areas (S1HL/S2) were activated. Cortical responses were also observed in the primary motor cortex (M1). Bilateral negative BOLD responses were measured in the caudate-putamen regions (CPu). In contrast to the cortical areas, activations in the CPu were only observed at TE = 30ms (Fig 4C, Table 2). These CPu responses were localized in the dorsolateral part of the CPu.

**Table 2:** Number of activated voxels as a function of the echo time (TE).

	TE = 30 ms	TE = 40 ms
Contralateral sensorimotor cortex	-	129 voxels
Ipsilateral sensorimotor cortex	-	16 voxels
Contralateral sensorimotor thalamus	221 voxels	36 voxels
Ipsilateral sensorimotor thalamus	258 voxels	111 voxels
Contralateral caudate-putamen	9 voxels	-
Ipsilateral caudate-putamen	7 voxels	-

Voxels (0.4 * 0.4 * 1mm^3^) were calculated based on clusters derived from group analysis results.

## Discussion

In the present study, BOLD functional activations in motor and sensory cortical and subcortical structures such as thalamus and caudate-putamen regions in response to a non-noxious sensorimotor electrical stimulation were shown. To our knowledge, studies exploring the sensorimotor network using stimulation-induced fMRI in rodents have either been performed using direct nerve stimulation [35] or direct cortical stimulation [40]. Studies using subcutaneous paw electrical stimulation have only shown activation in the primary somatosensory cortex [10,12–17,19–22] and rarely in the sensory part of the thalamus [11]. Here, a calibrated BOLD fMRI protocol was developed to explore the sensorimotor network by combining three main methodological approaches, namely a low level anesthesia, a non-noxious sensorimotor stimulation and a signal to noise preservation, that are discussed below.

For a given magnetic field strength, the temporal signal to noise ratio (tSNR) and the echo time (TE) are the parameters effecting BOLD sensitivity the most [37]. In the present work, these two parameters were controlled and optimized for cortical and subcortical brain regions. The maximum BOLD contrast change occurs when the TE is set to be equal to the T2* of the brain tissue being investigated. The measured T2* time in cortical areas was higher than in subcortical areas (Fig 1A). This is probably due to regional differences in the vascular system organization between the cortex and the subcortex [28] as well as the susceptibility effects. Consequently, two separate fMRI runs with two different TE were acquired for cortical and subcortical areas.

Moreover, based on the minimum required tSNR [31] in our fMRI experimental design, BOLD sensitivity maps were constructed according to an expected activation-induced signal change of at least 1%. Regions with a low tSNR were identified and acquisition parameters (SNR, resolution and echo position) were calibrated to improve BOLD signal change detection. The use of a two-shot GE-EPI with a properly set TE and a relatively low spatial resolution (0.4 mm^2^) resulted in sufficient spatial SNR values in all the regions of interest. Although the spatial SNR was lower in subcortical areas compared with cortical regions, due to the use of a surface coil in this study, the tSNR was sufficient enough to perform reliable BOLD mapping in both regions. BOLD sensitivity maps were also helpful to remove false positive activations at the stage of data analysis, as the analysis was restricted to brain regions with sufficient tSNR.

FMRI in rodents is most often performed under anesthesia. However, anesthesia directly affects the characteristics of the hemodynamic response, i.e. the shape of the hemodynamic response function (HRF) and influences the spatial specificity of BOLD signal [32]. Therefore, one must consider that for a specific anesthesia regimen, an appropriate HRF is required especially when dealing with heterogeneous brain structures. Isoflurane, the most used anesthetic in animal fMRI studies, was shown to have less effect on the shape of the HRF [32].Under low level isoflurane anesthesia, the “basis-SPM model” of HRF used in our study showed a high performance in the generation of statistical activation maps within heterogeneous brain structures. Consequently, our choice of isoflurane anesthesia regimen confirms its potential for stimulation-induced studies. Indeed, neurovascular responses to paw stimulation were reported under 1.1–1.3% isoflurane concentration [15]. However, these responses were smaller in intensity and spatial extension compared to medetomidine [16] or alphachloralose. This is probably due to an increase of the basal Cerebral Blood Flow (CBF), a consequence of the known vasodilatation effect caused by isoflurane at these concentrations. Nevertheless, no drastic effects on CBF were reported under 0.7% isoflurane anesthesia since the autoregulatory adjustment regime of the local CBF is still preserved under this concentration [41]. Furthermore, increasing isoflurane concentration leads to a reduction in BOLD responses to sensory stimulus but not in the neuronal evoked responses themselves [42]. This implies that the major limitation of isoflurane, at a high concentration level, in stimulation-induced BOLD responses is neurovascular uncoupling. Therefore, minimizing isoflurane anesthesia depth should limit vasodilatation, preserving the neurovascular coupling [13] and thereby BOLD detection. Moreover, to target specific pathways, i.e. sensorimotor pathways, neuroconnectivity of the related pathways should be preserved under anesthesia. Indeed, it has been reported that increasing isoflurane concentration in monkeys [43] reduces the extent of the functional brain connectivity. A resting-state rat brain study [44] showed that functional connectivity in cortical and subcortical pathways within the sensorimotor network was largely preserved under light and middle isoflurane anesthesia (0.5–1.0%). These results were also consistent with electrophysiological findings [45] in rats at similar isoflurane anesthesia concentrations.

In our study, a low isoflurane concentration (0.7–0.8%) was used to ensure a sedative effect and was combined with a soft muscle relaxation. Normal physiologic state in animals was maintained during the imaging experiment. Our BOLD measurements, in non-painful conditions, demonstrate that the presented soft isoflurane-anesthesia combined with a soft muscle relaxation may be an alternative protocol to preserve the neurovascular coupling as well as neuroconnectivity between the cortex and the subcortex.

Apart from the above anesthetic conditions, owing to our goal to elicit BOLD responses in both cortical and subcortical sensorimotor pathways, stimulation parameters were optimized in order to deliver sensorimotor excitations instead of the widely used sensory input. Indeed, the majority of studies dealing with BOLD responses induced by rat paw stimulation reported a common use of a short current pulse (~0.3 ms), with a variable stimulus frequency (~1–12 Hz) depending on the anesthetics used [10,11,17]. These stimulation protocols lead to BOLD induced-activations within the sensory cortex and usually did not extend beyond it. The use of short pulse duration (in non-noxious conditions) could be one explanation for the absence of responses that could be extended to subcortical structures. Van Camp et al. [23] reported that switching pulse duration of an electrical stimulation from 0.3 ms to 10 ms alters the characteristic of the input as well as the optimal frequency of the neuronal response. Moreover, it has been reported that the pulse duration parameter affects the selectivity of neural stimulation [46] and thereby the activated neuronal pathways [23].The optimal frequency stimulation allowing the largest BOLD response was found to be 8 Hz when 10 ms pulse duration was applied, under alphachloralose anesthesia [23]. This is illustrated in our experiments using optimized stimulation parameters [1.7 mA; 8 Hz; 10 ms], where sensory and proprioceptive pathways were recruited (see below), enabling cortical and subcortical sensorimotor activations (Fig 4).

**Fig 5:**
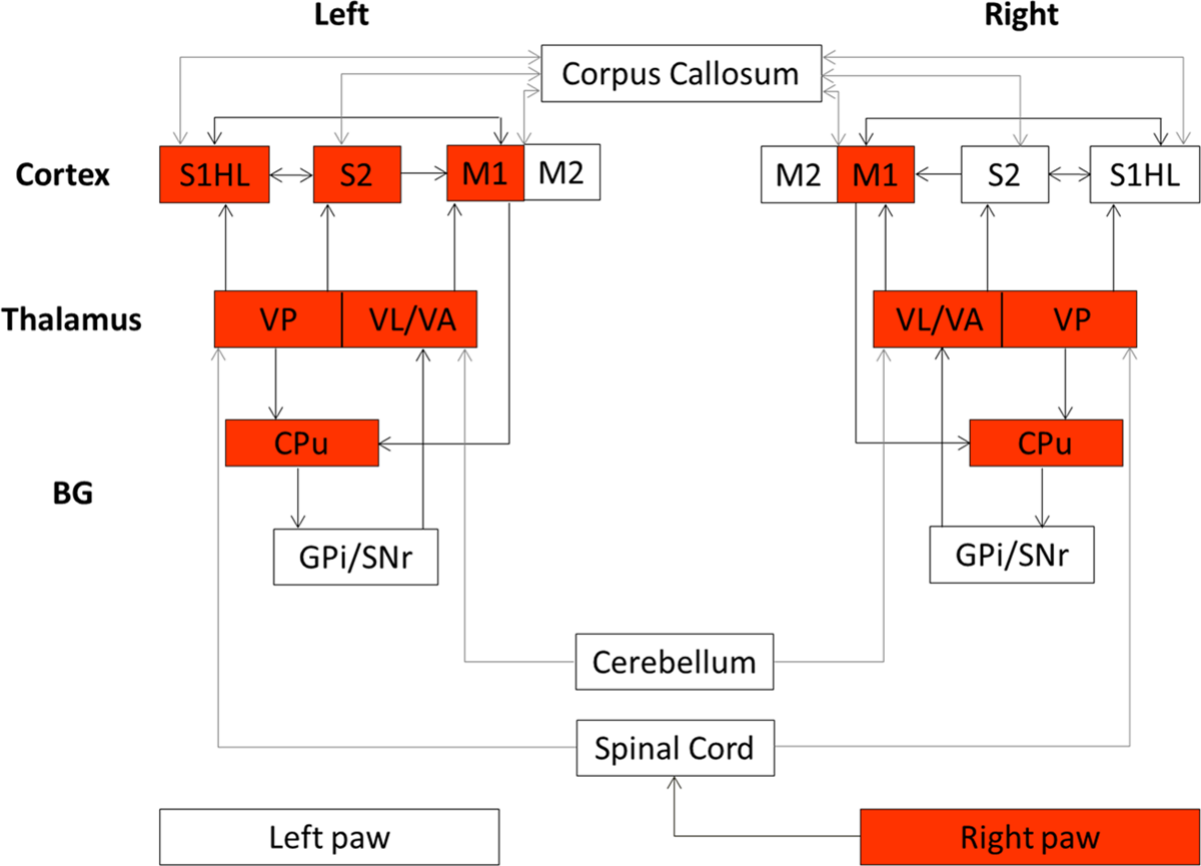
The rat brain sensorimotor network. A simplified description derived from previous studies [1–7]. Both brain hemispheres are presented and arrows represent connections. Cortical regions include the hindpaw primary/secondary somatosensory cortex (S1HL/S2) and the primary /secondary motor cortex (M1/M2). Subcortical regions include the thalamus represented by the posteroventral thalamic nucleus (VP), the motor thalamic nucleus including the ventral lateral thalamic nucleus (VL) and the ventral anterior thalamic nucleus (VA) and Basal Ganglia (BG) represented by the caudate putamen (CPu), the substantia nigra-pars reticulata (SNr) and the internal segment of the globus pallidus (GPi). Red boxes highlight the elicited structures after unilateral sensorimotor hindpaw stimulation in the present study.

Electrical stimulation resulted in simultaneous muscle and joint movements in all hindpaw’s digits (S1 Video). This indicates that stimulation inputs to the hindpaw could induce these movements. Joint and muscle afferent projections are extended to cortical and subcortical areas within the sensorimotor network of the rat brain [26,47] (Fig 5). Subcutaneous electrical stimulation probably activated Aβfibers from cutaneous receptors [25], while proprioceptive inputs, resulting from these digits movements, activated Aα fibers from joint and muscle spindle receptors [26,48]. These ascending fibers, of the proprioceptive afferents from the hindpaw, terminate on neurons in the dorsal nucleus of the spinal cord [49] which send axons to the cerebellum and to the contralateral thalamus. Peripheral sensory-proprioceptive inputs can directly elicit motor responses via spinal and cerebellar mediated reflex loops or via a supraspinal control. In this study, our adjusted electrical stimulation elicited BOLD activations at cortical and subcortical levels confirming the activation of supraspinal structures namely S1, S2, M1, VPL, VA, VL and CPu (Fig 4). These results do not exclude spinal and cerebellar responses [24,25], two prerequisite structures relay that are not in the scope of the present work.

Mapping studies [47] provided evidence on the proprioceptive functions of the lateral part of the VP nucleus, i.e. the VPL nucleus (Fig 4), where proprioceptive neurons from the hindpaw project most densely to the rostral part of the VPL. Activation is then released from this thalamic nucleus to the primary somatosensory cortex (S1) where the dysgranular portion of S1 (Fig 4) is devoted to proprioception. This somatosensory area provides the major activating input to the motor cortex, where the M1 and the M2 are interconnected with this dysgranular (and granular) part of S1 contributing to sensorimotor processing. The M1 receives also other reciprocal inputs from the motor thalamic nucleus, i.e. the complex VA/VL bilaterally activated in the present work (Fig 4). The VA/VL sends also projections to the CPu and plays a role of modulator of the cortico-striatal circuitry through multiple routes [50].

It is noteworthy that the elicited thalamic positive activations were bilaterally observed, in the present study. This is probably due to the fact that peripheral inputs are released through the spinoreticular tract cells that project to bilateral thalamic nuclei in both hemispheres [26]. Moreover, cortical primary motor areas were also bilaterally activated (although the contralateral activation was more pronounced), while the somatosensory activations (S1 and S2) tend to favor the contralateral hemisphere. The bilateral processing of motor information confirms that the functional neuroconnectivity across hemispheres in the motor network was preserved in our experimental conditions. Such motor responses could not be reached using a pure sensory stimulation and highlight the potential use of our refined stimulation parameters set. The dorsolateral part of the caudate-putamen (CPu) nuclei was also bilaterally negatively activated in the present work (Fig 4) and could be the result of two competitive pathways: the thalamo-cortico-striatal and the thalamo-striatal pathways. Strong connections exist between the motor cortex and the CPu structures [6] and the thalamo-cortico-striatal could be involved in the CPu response. Nevertheless, tract-tracing studies [1,2] revealed direct sensory inputs from the VP thalamic nucleus to the dorsolateral part of the CPu. The exact role of this projection has not yet been identified. However, with the presented results, we support the hypothesis that the VP thalamic nucleus, as well as the motor cortex, could provide direct sensory information to the CPu, required to sensorimotor control. Consequently, the VP thalamic nucleus is probably a sensorimotor structure rather than a sensory relay nucleus in the sensorimotor rat brain network. Unfortunately, due to the two acquired separate fMRI sessions (TE of 30 ms and TE of 40 ms), we were unable to address an exact functional causality of the CPu measured response.

In the present study, unilateral non-noxious sensorimotor electrical hindpaw stimulation resulted in robust positive and negative BOLD activations in the rat brain. The BOLD signal is dependent on complex interplay between cerebral blood flow (CBF), cerebral blood volume (CBV), and the cerebral metabolic rate of oxygen (CMRO2) [51], which are all related to neuronal activity. However, the mechanisms underlying neurovascular coupling in BOLD fMRI activations remain to be clarified as testified by the huge undergoing studies combining fMRI and direct neuronal recording [52] and those dealing with regional dependence of this coupling [27,53]. Indeed, while the positive BOLD response has been shown to be related to increased neuronal activity [54] accompanied by hemodynamic changes like increases in CBF and CBV, recent study by O’Herron et al. [55] by sampling vascular responses over the full range of a stimulus and measuring directly synaptic and spiking activities on the cat visual cortex, reported a partial decoupling between vascular and local neural signals. On the other hand, the mechanisms leading to negative BOLD response remain less well understood with studies questioning whether the relationship between neuronal activity and fMRI signals would be similar for cortical and subcortical structures, such as the caudate–putamen and the thalamus [27,53]. Results from Schridde et al. [56] during seizure in rat and Mishra et al. [57] in the rat model of epilepsy show negative BOLD signals despite an increase in neuronal activity in caudate putamen; calling therefore for caution when interpreting fMRI signals in both health and disease from the caudate–putamen, as well as possibly from other subcortical structures.

In the present study, by making assumption that neurovascular coupling hold within the sensorimotor network, we attributed the observed BOLD activations to neuronal activities induced by our tuned sensorimotor stimulation. Studies combining fMRI and direct neuronal measurements should give more insights into neurovascular coupling in the cortico-basal network.

## Conclusion

A calibrated BOLD fMRI protocol based mainly on a combination of a soft isoflurane anesthesia, a refined electrical stimulation and improvement of temporal SNR is presented. This protocol allowed BOLD measurements in cortical and subcortical areas of the sensorimotor network within and across brain hemispheres, suggesting the potential use of BOLD fMRI in the investigation of cortico-basal networks disorders in a rodent model.

## Supporting information

S1 Video Illustration of movements of the rat hindpaw’s digits in response to the electrical stimulation. (AVI)

S1 Table Cardiac frequency, current intensity stimulation. (XLSX)
